# Within-season variability of fighting behaviour in an Australian alpine grasshopper

**DOI:** 10.1371/journal.pone.0171697

**Published:** 2017-04-12

**Authors:** Giselle Muschett, Kate D. L. Umbers, Marie E. Herberstein

**Affiliations:** 1 Department of Biological Sciences, Macquarie University, North Ryde, NSW, Australia; 2 School of Science & Health, Western Sydney University, Hawkesbury, Richmond NSW, Australia; Fundacao Oswaldo Cruz, BRAZIL

## Abstract

Throughout the breeding season, changing environmental and biological conditions can lead to variation in the reproductive landscape of many species. In alpine environments temperature is a key driver of behaviour for small ectotherms such as insects, but variable biotic factors such as mate quality and availability can also influence behaviour. *Kosicuscola tristis* is a small semelparous grasshopper of the Australian alpine region. In a rare behaviour among grasshoppers, *K*. *tristis* males engage in vigorous fights over access to females, involving mandible displays, kicking, biting and grappling. In this study we describe the variation in fighting behaviour of *K*. *tristis* throughout the breeding season and test several hypotheses related to temperature, body size, mating behaviour, and female quality. We show that *K*. *tristis* males are more aggressive toward each other at the end of the breeding season than at the beginning. This increased aggression is associated with decreased daily average temperatures (from ~20°C to ~9°C), decreased mating activity, increased female fecundity, and an unexpected trend toward an increase in female-to-male aggression. These results suggest that *K*. *tristis* is likely under increased selective pressure to time key life cycle events with favourable biological and climatic conditions. The stochastic nature of alpine environments combined with a relatively short life span and breeding season, as well as limited mating opportunities toward the end of the season may have contributed to the evolution of this extraordinary mating system.

## Introduction

Interactions between biotic and abiotic factors can lead to significant seasonal variation in the reproductive landscape of insects. Fluctuations in climatic conditions, particularly temperature, can result in peaks in mating activity during warmer months [1, 2, 3]. While warm temperatures are important for reproduction (e.g. [4, 5, 6]), the peak of mating activity does not track optimal temperature conditions in all species [1]. For example, in some grasshoppers (e.g. *Stenobothrus lineatus*), mating activity peaks in colder conditions early in the season and declines as the summer progresses [2]. This suggests that although abiotic factors are strongly influential, biotic factors also influence mating activity.

Biotic factors such as sex ratio, mate availability, mate quality, and age change over the course of mating seasons, and can affect male and female mating behaviour. For example, the early emergence of adult males, known as protandry, typically leads to male-biased sex ratios early in the breeding season [7, 8]. In *Photinus ignitus* fireflies, females are choosier at the beginning of the breeding season when the operational sex ratio is largely male-biased and the probability of loosing a mating opportunity is low [9]. In addition, protandrous species typically exhibit scramble competition mating systems, where competition among males is restricted to a race for mating opportunities with recently emerged unmated females [10, 11, 12]. However, as the breeding season progresses the quality of mates can vary greatly. Female fertility coupled with the effects of advanced age can affect reproductive success [13, 14]. For example, in the carabid beetle *Notiophilus biguttatus* egg production increases but egg size decreases with female age towards the end of the breeding season [15].

Seasonal variation in sex ratio, mate quality and age, can result in peaks of intra-sexual conflict where males compete with each other for access to females [16]. For example, as the summer progresses, the sex ratio in eastern amberwing dragonflies (*Perithemis tenera*) skews toward males as females either die or emigrate, leading to a greater frequency of male-male territorial fights and chases [17, 18]. Because fighting can be costly in terms of lost reproductive success, [19, 20, 21], increased energy expenditure [22], increased risk of predation [23, 24] and increased risk of injury or death [25, 26, 27], most species have developed mechanisms by which individuals avoid conflict [28]. In the Orthoptera for example, male crickets typically rely on acoustic signals to deter opponents rather than physical conflict [29]. Despite these ritualistic mechanisms, there are circumstances under which fighting will still take place.

Australian chameleon grasshopper (*Kosciuscola tristis*) males frequently engage in physical combat over access to females [30, 31], a rare behaviour among grasshoppers [32, 33, 34]. As an alpine specialist, *K*. *tristis* experiences substantial fluctuations in weather conditions, which can vary from 0°C in late autumn to over 25°C in summer (http://www.bom.gov.au/climate/data/index). Like many other alpine invertebrates, *K*. *tristis* is semelparous, having only a single, relatively short breeding season (mid January through early May [35]). Male chameleon grasshoppers compete for fertilizations in at least two ways, either by engaging in physical fighting or via sperm competition. Male-male fights in *K*. *tristis* typically occur over access to ovipositing females [30]. A male will usually mount an ovipositing female and remain on her dorsum while she oviposits. When a rival tries to displace a mounted male a fight ensues, usually involving mandible displays, biting, kicking and grappling [30]. While male *K*. *tristis* lack weapons, physical combat is energetically costly and frequently leads to injury [30, 31]. Evidence suggests that contest outcome in *K*. *tristis* is not determined by body size but by comparative male brightness, and brighter males win contests [31]. Under laboratory conditions, *K*. *tristis* females will readily mate multiply (Muschett unpub. data) suggesting the possibility of sperm competition, but the mechanisms of sperm precedence in this species are unknown. As in many grasshoppers, *K*. *tristis* has a single spermatheca at the end of a long coiled spermathecal duct [36] (Muschett unpub. data]. Yet despite this shared spermathecal structure, sperm precedence can vary widely between grasshopper species [37, 38].

The aim of this study was first, to describe within-season variability fighting behaviour in *Kosciuscola tristis* and, second to assess which factors are associated with this variation. To measure fighting behaviour across the season we recorded the number and type of aggressive behaviours between individuals. To assess the potential drivers of the variation in fighting behaviour we measured: 1) temperature, 2) mating duration, 3) latency to mate, 4) female quality (i.e. fecundity as indicated by mature oocyte number and weight), 5) female aggression, and 6) male body size and 7) female body size. We chose these factors as they typically influence male investment and fighting intensity [39, 40]. We hypothesized that as the breeding season progresses fighting behaviour changes, as the perceived value of limited mating opportunities increases. We predicted that the propensity to fight and fighting intensity would peak at the end of the breeding season [41].

## Materials and methods

### 1. Study species

The chameleon grasshopper *K*. *tristis* is endemic to the Australian alpine region, regularly found above 1800m across the highest peaks—from Mt. Kosciuszko in New South Wales, to Mt Hotham in Victoria [42, 43, 44]. It is a small, flightless member of the Acrididae, and is hyper-abundant at the height of its season (March through April). Similar to other alpine species, *K*. *tristis* has an annual life cycle with only one generation per year [31, 45, 46]. Nymphs emerge in early November, while adults begin to appear in mid-January. Females oviposit on small patches of bare soil from mid March through early May. The adults die off in winter and the population overwinters as eggs ([42, 45, 46], K. Umbers, pers. obs.).

### 2. Temperature across the season

We obtained monthly mean minimum and maximum temperatures from the Bureau of Meteorology of Australia Thredbo Top Station (station number 071032) for the 2013–2014 growing/breeding season (http://www.bom.gov.au/climate/data/index) (S1 Data). We used two indicators of season length. One indicator uses the first day above 11°C as an indicator of the start of the season [47]. The second indicator was estimated using the annual sum of days above 13°C based on the embryonic development of European species of Acrididae [48]. Since there are no studies on the embryonic development of *K*. *tristis* the index presented here is used as a general guideline only and results must be interpreted with caution.

### 3. Specimen collection and behavioural trials

We collected grasshoppers from Dead Horse Gap trail in Kosciuszko National Park, Thredbo, NSW, Australia (36° 50’ 21.0” S, 148° 27’ 85.3” E), at 1958 m in altitude. In order to sample different periods during the breeding season we carried out behavioural assays on three separate occasions during the summer of 2014: 1) 29 January– 4 February, 2) 10–16 March and 3) 4–10 April, referred to as Periods 1, 2 and 3, respectively. Males and females were kept in separate large mesh enclosures (69 x 69 x 122 cm), each of which contained either potted or naturally collected tall sedges (*Carex* sp.). The enclosures were kept at ambient temperature and daily light cycles. Food intake was monitored daily and replaced as necessary, and the enclosures were sprayed with ample water three times a day. The day after collection, males were individually marked using bee tags (Pender’s Beekeeping Supplies) fixed to their pronotum [31] or non-toxic paint pens (Uni-POSCA™). Males and females were kept separate for at least 48 h prior to behavioural trials, a common practice when assessing aggressive behaviour in Orthopterans [31, 49, 50]. Isolation provides individuals the opportunity to recover from previous aggressive and mating experiences [49]. The experimental arenas consisted of a plastic box (40 x 30 x 20 cm) with mesh sides. Inside each box we placed common vegetation collected from the field to serve as a substrate, mainly dead snow grass (*Poa hiemata*) and alpine grevillea (*Grevillea australis*) [31]. To eliminate chemical cues, experimental arenas were emptied and cleaned with 70% ethanol at the end of each trial.

Behavioural trials were conducted between 48 and 72 hours after initial collection. We carried out trials where five males competed for one female [31]. This number of males is within the range observed fighting over a female under natural conditions, with groups ranging from two to six males [30]. On the day of the trial, we haphazardly selected five males out of the all-male enclosure, placed them in the experimental arena and allowed them to acclimate for 5 min. After the acclimatization period, an adult female was selected from the all-female enclosure and introduced into the centre of the arena. Each trial was run for 60 min and the behaviour of each individual recorded (see *Fighting behaviour*). Each individual grasshopper was only used in one behavioural trial.

### 4. Fighting behaviour

We carried out *n* = 55 behavioural trials throughout the breeding season: *n* = 18 trials in Period 1 (90 males, 18 females), *n* = 20 trials in Period 2 (100 males, 20 females) and *n* = 17 trials in Period 3 (85 males, 17 females) for a total of 275 males and 55 females. Because *K*. *tristis* is hyper-abundant at this time, it is unlikely sampling efforts affected sex ratios. All observers had prior experience recording grasshopper behaviour. Aggressive interactions typically occur in short bouts between pairs of individuals, or between a mounted male and a rival. This aspect of *K*. *tristis* behaviour made recording aggressive interactions particularly straightforward.

A typical fight between *K*. *tristis* males can involve a mandible flare display (or a series of mandible flares), followed by a male mounting an opponent, where bites, kicks or grapples can ensue [31] (Table 1). Among these behaviours, grapples are the most energetically costly [51, 52]. However, when a male mounts another male, grappling and biting do not always occur and two additional behaviours can be observed: the aggressor may either remain perched on his opponent and/or he may attempt to mate with his opponent. Grapples and mating attempts only occur when males mount each other. Because female grasshoppers can show aggression towards males [53, 54], we also recorded any instance of female-male aggression. Female aggressive behaviours are limited to kicks and grapples (Table 1). We recorded the frequency of each aggressive behaviour in each trial.

**Table 1:** Aggressive behaviours in grasshopper behavioural trials

Behaviour	Description	Observed in
Mandible flare	Male raises himself on forelegs and hyperextends the mandible, while shaking its head shaking side to side, wiggling and flattening its antennae laterally.	Males only
Mount	Male mounts another male, aligning himself with the anterior-posterior axis of opponent. A male must remain mounted for more than 30 sec to constitute riding.	Males only
Mating attempt	A riding male curves his abdomen under abdomen of ridden male, epiphallus is visible	Males only
Kick	Quick movement of either or both hind legs, tibia rushes backwards and/or upwards	Males and females
Bite	Pinches opponent with mandibles	Males and females
Grapple	Repeatedly pushes or pulls opponent mainly with cephalic and medial femora, hind femora/tibia may also be used	Males and females

### 5. Mating behaviour

To describe mating behaviour during the trials, we recorded latency to mate and mating duration. Latency to mate was measured from the time the female was introduced into the experimental arena until a male mounted a female and copulation began (i.e. a male curved his abdomen beneath the female’s and connecting their genitalia). The first male to mount a female did not always copulate. Mating was measured from the time copulation began until a male and female disengaged their genitalia, with the male typically walking away from female.

### 6. Body size measurements and female dissections

Although *K*. *tristis* is univoltine and has only one generation per year, size at maturity of each individual can be highly plastic. A within-season variation in size could reveal the presence of a late season cohort [55, 56, 57]. Body size measurements (i.e. femur and pronotum length) were carried out post-hoc in order to minimize handling. After each trial, males were placed in a freezer (-20°C) for 48h and preserved in 70% ethyl alcohol. Following [58] we measured femur and pronotum length using Vertex™ Vernier digital callipers (England, UK) to the nearest 0.01 mm. Femur length was taken from the trochanter to the beginning of the femur-tibia joint. Pronotum length was measured along the median carina, from the prescutum to the hind margin. Females were equally treated and measured, but were weighed before being preserved in 70% ethanol. To determine if female weight varied over the season, they were weighed to the nearest 1 mg using a Jscale^TM^ (Pheonix Arizona, USA).

We used the number and weight of mature oocytes as an indicator of female fecundity. We measured mature oocyte number and not number of eggs laid because the mortality rate of *K*. *tristis* in captivity is very high [31]. It is likely most females would have died before ovipositing, leading to a loss of data. Females were dissected dorsally by making an incision from the epiproct to the pronotum following the medial carina, exposing the reproductive system. In grasshoppers the ovaries are paired and consist of many tubular ovarioles, attached by the pedicel to two lateral oviducts [59, 36]. Ovarioles contain progressively developed eggs or mature oocytes with the largest and most developed oocytes located at the distal end, nearest to the oviduct. We also checked females for parasites, mainly Diptera and Nematoda larvae [60]. In order to count eggs and identify parasites we used a stereoscope at 10 × magnification.

### 7. Data analysis

All analyses were carried out in R v2.15.1 [61]. Data are presented as mean ± SD unless stated otherwise. We present the analysis for each question, below.

#### 7.1 Is there within-season variation in male and female aggression and mating behaviour?

To compare the number of male-male fighting behaviours and female-male fighting behaviours per trial in Periods 1, 2 and 3, we used generalized linear models (GLM) with aggression as the response variable and the different periods (P1, P2, P3) as the explanatory variables. To determine which aggressive behaviours were more common in Period 1, 2 and 3 we performed a Pearson’s Chi-square test based on 9999 Monte-Carlo re-samplings (“stats” package). To assess variation in latency to mate and mating duration in Period 1, 2 and 3 we used a Kruskal-Wallis rank sum test, with a posteriori Conover-Iman test.

#### 7.2 Is there within-season variation in male and female size and female fecundity?

**Fig 1:**
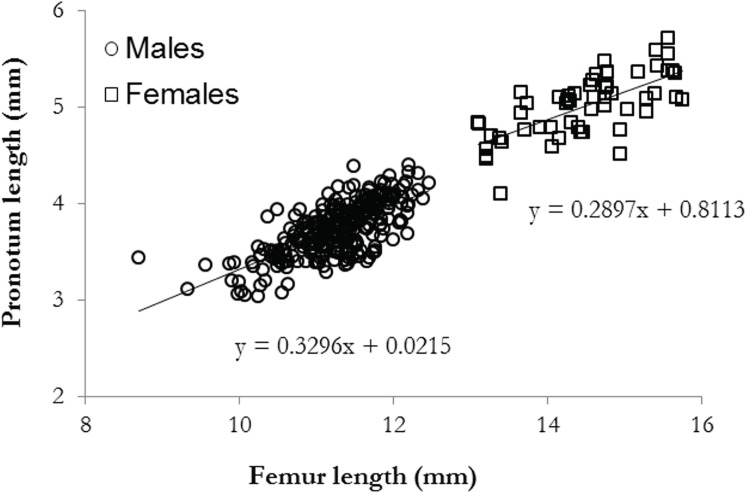
Pearson correlation between *K*. *tristis* femur and pronotum length. Pearson correlation between male (open circles) and female (open squares) pronotum and femur length.

As per [62], a preliminary analysis showed there was a strong correlation between pronotum and femur length in both males and females (Male: Pearson’s r = 0.64, *n* = 275, *P* = 0.02; Female: Pearson’s r = 0.56, *n* = 55, *P* < 0.01; Fig 1). Therefore, femur length was used as a proxy for body size. We used the Kruskal-Wallis rank sum test, with a posteriori Conover-Iman test to determine within-season variation in mean male size and female body size per trial. The same test was used to determine variation in female weight, egg number and egg weight over the season.

#### 7.3 Which factors explain within-season variation in male-male aggressive behaviour?

We used a Principal Component Analysis (PCA) to determine the relationship between variation in male aggression with (a) temperature (days above 13°C), (b) mating duration, (c) latency to mate, (d) female fecundity (egg weight) (e) female aggression (f) mean male body size. Female body size was not included as a variable because a preliminary analysis showed that female body size did not vary throughout the season (femur length: P1, $\bar{x}$ = 14.96, ± 0.54; P2, $\bar{x}$ = 14.44, ± 0.83, P3, $\bar{x}$ = 13.99, ± 0.61). We used the PCA to determine which variables were the most relevant, or best explain our response variable. In other words, it was used to reduce the number of variables to those that summarize the original information, and reveal patterns the distribution of data points that could not be found by analysing each variable separately. In the PCA the axes of a biplot are a pair of principal components. These axes are labelled PC1, PC2, and so on. It uses points to represent the scores of the observations on the principal components, and it uses vectors to represent the coefficients of the variables on the principal components [63]. Once we determined which variables were the most relevant, we used generalized linear models (GLM) with male aggression as the response variable and temperature (days above 13°C), female egg weight, and female aggression (the total number of kicks and grapples per female per trial) as the explanatory variables.

#### 7.4 Is there a relationship between female-male aggression and mating behaviour?

We used a Spearman’s rank correlation coefficient to assess whether there was a relationship between female-male aggression and mating duration across the season.

#### Ethics statement

No specific permits were required for the described field studies however, we did attain permits from New South Wales National Parks and Wildlife Service for collecting *Kosciuscola* grasshoppers in Kosciuszko National Park (Scientific License number S12256).

## Results

### 1. Temperature across the season

We estimated the start of the season in October 2013, which registered the first day above 11°C. Season length was 104 days. Nymphs begin to emerge in early November and adults eclose in early to mid-January ([42, 45, 46], K. Umbers, pers. obs.). We carried out our trials from 30 January to 14 April 2014 to increase the likelihood of sampling an adult population. During our trials, Period 1 had the highest mean number of days above 13°C (25 d), while Period 3 only had two days above 13°C (Table 2).

**Table 2:** Mean monthly minimum and maximum temperatures for the 2013–2014 Australian Spring, Summer and Autumn.

Year	Month	Mean Min T (°C)	Mean Max T (°C)	No. of days above 13°C
2013	October	N/D	8.0	3
2013	November	1.4	10.9	10
2013	December	4.9	14.7	21
2014	January	7.4	18.1	23
2014	February *	7.5	17.6	25*
2014	March *	5.5	12.9	19*
2014	April *	2.3	9	2*
2014	May	- 0.2	5.8	0

N/D = no data available.

* indicates months when behavioural trials were carried out.

### 2. Behavioural trials

**Fig 2:**
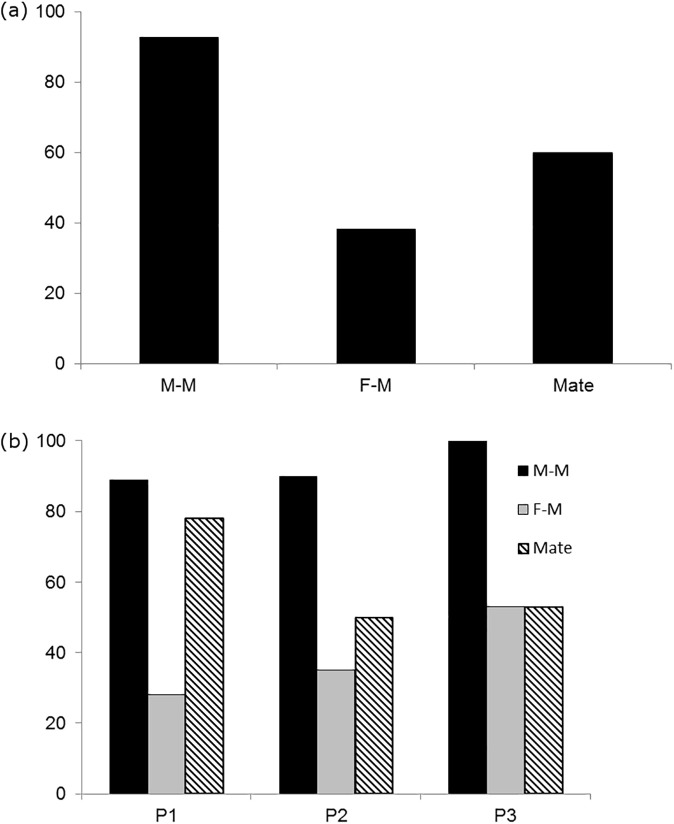
Percentage of trials with aggression and mating. Percentage of trials (*n* = 55) with male-male aggression, female-male aggression and mating (Fig 2A), and percentage of trials with male-male aggression, female-male aggression and mating per sampling period; P1, *n* = 18; P2, *n* = 20; P3, *n* = 17 (Fig 2B). M-M indicates male-male aggression, F-M indicates female-male aggression, Mate indicates mating.

The percentage of trials with male-male aggression was higher than those with female-male aggression or mating (Fig 2A) (S2 Data, S3 Data, S4 Data). As the season progressed, the percentage of trials with male-male and female-male aggression increased slightly, while the percentage of trials where mating occurred appeared to peak at the beginning of the season (Fig 2B).

### 2.1 Is there within-season variation in male and female aggression and mating behaviour?

The GLM showed that the variation in male-male aggression was explained by the advancing breeding season, and males were more aggressive at the end of the breeding (P3) than at any other time during the season (P1, P2) (Table 3). The frequency of certain aggressive behaviours also varied throughout the season (Table 4). Males mounted, grappled, and bit other males more often in Period 3 than in Period 2 or Period 1 (Table 4). There were fewer male-male mating attempts in Period 2 than in Period 1 or Period 3 (Table 4).

**Table 3:** Estimates for GLM models describing the relationship between male-male aggression and the different sampling periods throughout the breeding season.

	Estimate	SE	Z	*P*
P1 –P3	-1.44	0.23	-6.32	2.5e-10*
P2 –P3	1.64	0.24	6.92	4.5e-12*
P2 –P1	0.19	0.30	0.65	0.52

**Table 4:** Differences in the frequency of male-male aggressive behaviours per trial between Period 1, Period 2 and Period 3.

Aggressive Behaviour	Sampling Period			Pearson’s *χ*^2^	*P*—value
	P1	P2	P3		
Mandible flare	18	9	13	3.02	0.20
Mount	25	17	73	47.86	< 0.00*
Mating attempt	9	1	10	7.30	0.02*
Kick	3	5	7	1.60	0.50
Bite	0	0	4	8.00	0.04*
Grapple	6	2	19	16.00	< 0.00*

**Fig 3:**
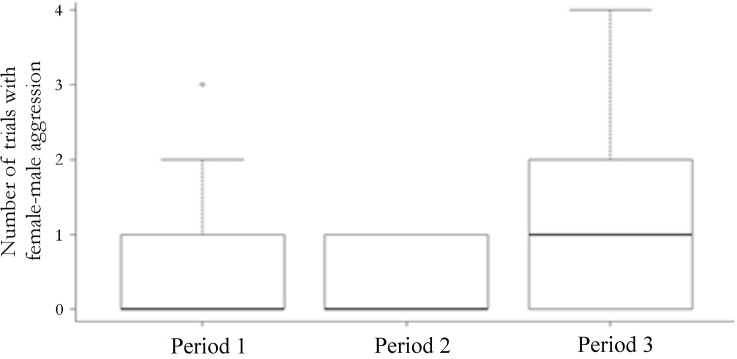
Within-season variability in trials with *K*. *tristis* female-male aggression (kicks and grapples). Data are presented in box plots, depicting the median value (solid horizontal line), 25^th^ and 75^th^ percentile (box outline), 90^th^ percentile (bars above boxes), and outliers (open circle).

Of the 35 observations of female-male aggression, 77% (*n* = 27) were kicks and 23% (*n* = 8) were grapples, no other aggressive behaviours were observed. In these instances, aggression was directed at males that approached females from behind within 1 cm (11 observations, 31%) or males that mounted females (24 observations, 69%). The GLM showed that the variation in female-male aggression was explained by the advancing breeding season, and females were more aggressive at the end of the breeding (P3) than at any other time during the season (Table 5; Fig 3).

**Table 5:** Estimates for GLM models describing the relationship between female-male aggression and the different sampling periods throughout the breeding season.

	Estimate	SE	Z	*P*
P1 –P3	-0.97	0.42	-2.33	0.02*
P2 –P3	-1.21	0.44	-2.76	< 0.01*
P2 –P1	-0.24	0.52	-0.46	0.644

**Fig 4:**
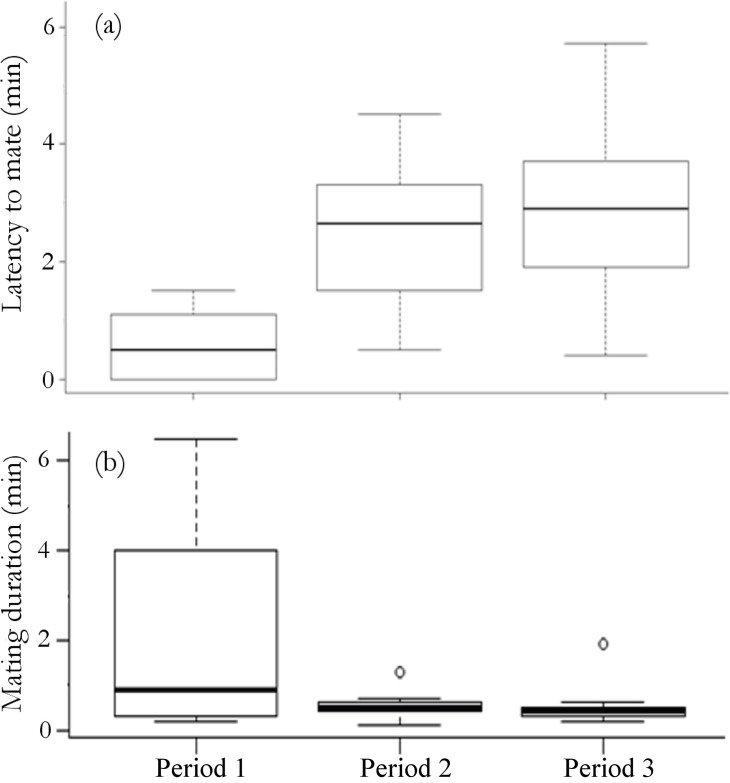
Within season variability in mating behaviour. Within season variability in **(a)** latency to mate and **(b)** mating duration during *K*. *tristis* behavioural trials across the breeding season. Data are presented in box plots, depicting the median value (solid horizontal line), 25^th^ and 75^th^ percentile (box outline), 90^th^ percentile (bars above boxes), and outliers (open circle).

Latency to mate varied across the season (Kruskal Wallis H_2_ = 15.36, *P* = 0.0005), with a shorter latency early in the season (*P* < 0.01; Fig 4A) (S5 Data). Mating duration was more variable in Period 1 than in Period 2 and Period 3, but the overall the median duration was not significantly different across the season (Kruskal Wallis H_2_ = 4.70, *P* = 0.09; Fig 4B).

#### 2.2 Is there within-season variation in male and female size, female fecundity and female rates of parasitism?

Male femur size ranged from 8.70 mm to 12.47 mm (11.27 ± 0.56 mm, *n* = 275; Table 6). Male size varied throughout the season (Kruskal-Wallis H_2_ = 7.8348, *P* = 0.0195) (S6 Data). Males in Period 2 were smaller than males in Period 1 (*P* = 0.01) but there were no size differences between Period 2 and Period 3 (*P* = 0.41) or between Period 1 and Period 3 (*P* = 0.49). Female femur size ranged from 13.1 to 15.8 mm (14.46 ± 0.67 mm, *n* = 55; Table 6). Female size also varied throughout the season (Kruskal-Wallis H_2_ = 14.04, *P* = 0.0008), and females were smaller later in the season (*P* = 0.0002). Parasitism rates were low, and only two of 55 females (4%) had parasites upon dissection: one nematode worm and one Diptera larvae, respectively.

**Table 6:** Mean ± SD of *K. tristis* male and female femur length (mm) and female weight (mg) across the season.

	Male		Female		
	Femur (mm)	*n*	Femur (mm)	Weight (mg)	*n*
**Period 1**	11.3 ± 0.5	90	15.0 ± 0.6	591 ± 85.0	18
**Period 2**	11.1 ± 0.6	100	14.4 ± 0.8	686 ± 95.8	20
**Period 3**	11.3 ± 0.6	85	14.0 ± 0.6	687 ± 87.4	17

Female weight ranged from 486 to 865 mg ($\bar{x}$ = 643.7 ± 95.7 mg, *n* = 55) (S7 Data). There were overall significant differences in female weight across the season (Kruskal-Wallis H_2_ = 11.49, *P* = 0.003). Females were heavier in Period 2 (*P* = 0.004) and Period 3 (*P* = 0.005) than in Period 1. Upon dissection, *n* = 39 females had mature eggs and egg number ranged from 12 to 31 ($\bar{x}$ = 16 ± 4.4) (S8 Data). Collectively, the weight of mature eggs per female ranged from 48 to 248 mg ($\bar{x}$ = 102.1 ± 75.3 mg, *n* = 39) and varied throughout the season (Kruskal-Wallis H_2_ = 31.59, *P* < 0.01). Mature egg weight was higher in Periods 2 and 3 (*P* < 0.01) than in Period 1 (S9 Data). In Period 2 and Period 3 mature eggs accounted for 21% to 22% of a female’s body weight, respectively.

#### 2.3 Is there a relationship between female aggressive behaviour and mating behaviour?

**Fig 5:**
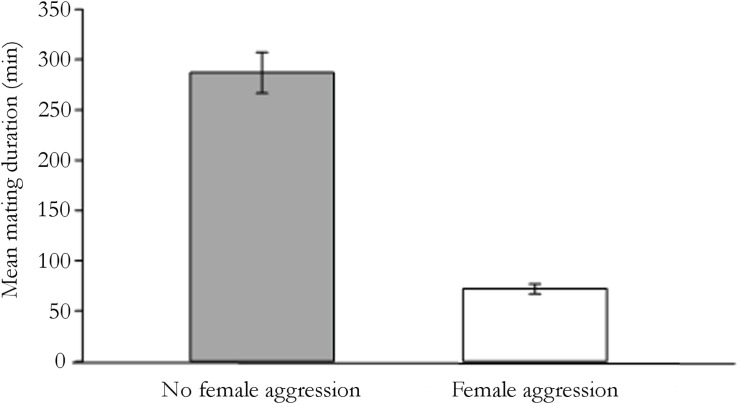
Variability in mean mating duration and female aggression. Within season variability in mean mating duration in trials with and without female aggression.

Mating duration was significantly reduced in trials with female to male aggression compared to trials with no female-male aggression (r_s_ = -0.45, *n* = 41, *P* < 0.01; Fig 5).

#### 2.4 Which factors explain within-season variation in male-male aggressive behaviour?

**Fig 6:**
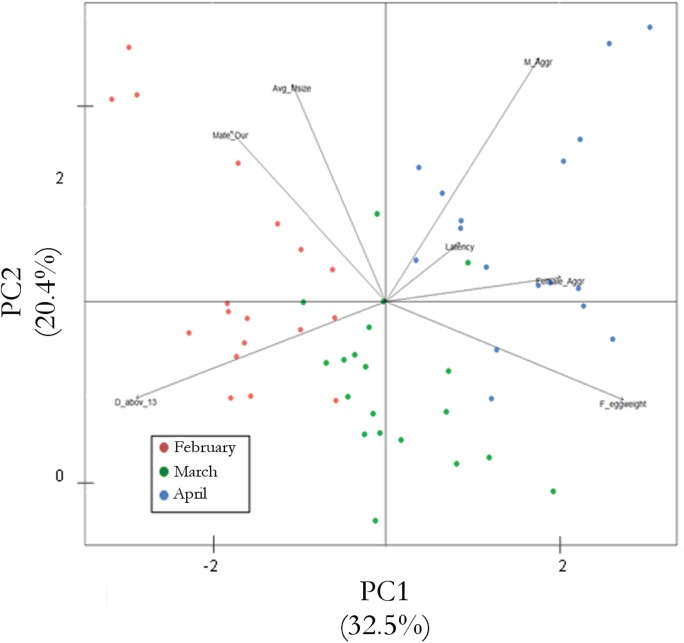
Principal component analysis (PCA). PCA of temperature (days above 13°C), average male size, male and female aggression, mating behaviour (latency to mate and mating duration), and female fecundity (egg weight). The plot shows each trial per month of the season as a coloured dot and each variable as a vector. Vectors that are close together are highly correlated, while vectors that are orthogonal are poorly correlated. Length and direction of arrows show the strength and direction of correlation, respectively.

A Principle Components Analysis (PCA) showed that three components explained 69% of the total variance in the aggression data the trials during Period 1, 2 and 3 (component 1: 32.5%, component 2: 20.4% and component 3: 15.8%). Using these three components the majority of trials grouped together according to the months of the season (Fig 6). The GLM showed that differences in temperature (days above 13°C), female to male aggression and egg weight accounted for a significant amount of variation in male aggression (Table 7).

**Table 7:** Estimates for GLM models describing the relationship between male aggression and temperature (Days above 13°C), female egg weight and female-male aggression in *Kosciuscola tristis*.

	Estimate	SE	Z	*P*
Intercept	2.65	0.18	14.42	< 2e-16 *
Days above 13°C	-0.06	0.01	-8.47	< 2e-16 *
Female egg weight	0	0	-3.77	0.00 *
Female-male aggression	0.44	0.13	3.38	0.00*

## Discussion

The aim of this study was to determine whether fighting behaviour in *K*. *tristis* varies across the breeding season and if so, what factors were associated with this variation. We found that male-male aggression increased as the season progressed and that towards the end of the season males engaged more often in grapples, the most vigorous and presumably the most energetically costly aggressive behaviours [51]. We also observed a trend that females were more aggressive toward males at the end of the season and that this may relate to mating duration. There are a number of abiotic and biotic factors that can explain the changes in mating and fighting behaviour we observed. These include: temperature, mating duration, latency to mate, female quality, female aggression and male size.

### Effect of temperature on mating and male–male aggression over the season

In alpine regions, small ectotherms such as insects are particularly susceptible to extreme fluctuations in climatic conditions, particularly temperature [64]. For example, mating activity in some species of grasshoppers increases during the warmer months in milder climates such as alpine regions (see [2] for review). In Thredbo, mild ~ 20°C summer temperatures give way to much cooler conditions, with average Autumn temperatures fluctuating around 9°C. (Table 2). Winter temperatures are too low for *K*. *tristis* to maintain normal metabolic function and at the onset of winter cooler temperatures push *K*. *tristis* to the edge of its cold tolerance capacity [44]. Like many other alpine invertebrates adult *K*. *tristis* are unable to enter a dormant state, and the first frosts typically lead to death [65]. As a result males may have fewer opportunities to secure a mate later in the breeding season and there is increased competition among males to secure diminishing mating opportunities at this time. Theory predicts that when environmental resources are so low that survival is almost impossible the optimal strategy is to invest fully in reproduction even if it leads to death, a concept commonly referred to as ‘terminal investment’ [66, 67]. Therefore, even though temperature and energetic resources may not be optimal at the end of the breeding season, male-male contests commonly escalate to physical combat.

Alternatively, the increase in male-male aggression toward the end of the breeding season could be due males that have had an opportunity to feed and improve their condition and are therefore more able to fight. In *Gryllus pennsylvanicus* crickets, heavier males are more aggressive than males that are lighter males [51]. However, due to the relatively short alpine breeding season it is likely there a trade off between optimal temperature development days and resource availability. It is possible that despite having had more opportunities to feed, males may not have had sufficient exposure to optimal temperatures in order to improve their condition.

### Female quality and mating behaviour

Our results suggest that females are more likely to have mature eggs at the end of the season than the beginning. It is likely that females change from an initial phase devoted mainly to mating with little or no egg development, to a later phase with lower mating activity but more egg development. Such variation in female fecundity may influence male fighting behaviour [40]. For example, male preference for larger, more fecund females in milkweed longhorn beetles (*Tetraopes tetraophthalmus*) results in aggressive displacement of smaller rival males [68]. The more fecund, late-season *K*. *tristis* females could be ‘higher reproductive value’ than early season females and elicit a more intense aggressive response from males to gain access to these females. However, we are aware that fecundity is best measured by number of eggs laid and not mature oocyte number. We had to rely on the latter measure because of the high mortality rate of *K*. *tristis* in captivity.

In general, the reproductive value of females changes throughout the season due mainly to the effects of varying fecundity and mating status. We expect male mating effort and male-male aggression to change accordingly [69, 70]. We found that females had a greater number of mature eggs towards the end of the season, and it was one of the main factors that explained the increase in male-male aggression at this time. At first this finding might seem counterintuitive since copulation started very quickly at the beginning of the season when females had no mature eggs. However, the significantly reduced latency to mate early in the season makes sense if mating induces egg development in *K*. *tristis* as it does in other grasshoppers [71]. For example, copulation induces rapid oocyte development in the desert locust *Schistocerca gregaria* [71], *Rhodnius prolixus* [72], and *Aedes aegypti* mosquitoes [73]. If copulation does induce oocyte development in *K*. *tristis*, we predict that unmated adult females do not develop any, or likely significantly fewer eggs. Based on our results however, we cannot rule out the possibility of egg development independent of copulation because we do not know the females’ prior mating history.

On the other hand, female mating status and sperm competition, in the form of sperm precedence, may also explain the increase in male-male aggression late in the season. *Kosciuscola tristis* females readily re-mate with different males (K. Umbers pers. obs., Muschett unpub. data), allowing for the possibility of sperm competition. Under first male sperm precedence we expect males to favour females that have never mated, while under last male precedence we expect higher degree of mate guarding by males [74]. Although we do not have direct evidence of the fertilization success of males in a competitive scenario, our behavioural data suggest possible mechanisms.

In *K*. *tristis*, males are regularly observed mounted on both sub-adult and ovipositing females, and males frequently engage in fierce fights over the latter [30]. In a system with high sperm competition, when a male is aware that he is the first mate (e.g. via chemical cues, [75]) we would expect the transfer of large ejaculate volumes given the high risk of their sperm being out-competed. In *K*. *tristis*, the shorter latency to mate early in the season coupled with longer mating durations (several > 60 min) could favour matings with recently eclosed, unmated adult females while transferring larger ejaculates, potentially filling the female’s spermatheca and preventing further inseminations. From literature on other species, extended mating duration leads to larger ejaculate volumes (e.g. stalk-eye fly (*Cyrtodiopsis whitei*) [76]; egg bug (*Phyllomorpha laciniata*) [77]; bruchid beetle (*Callosobruchus maculatus*) [78]). In grasshoppers the number of sperm in a female’s spermatheca also increases with mating duration in several species [79, 80, 81]. Conversely, in a system with last male sperm precedence we expect males to prefer to mate with females that are very close to ovipositing, and / or guard mates until oviposition [82]. Under this scenario, the longer copulations observed in *K*. *tristis* early in the season could be a form of mate guarding, maintaining contact long after insemination is complete, preventing inseminations by rival males [83].

As in many grasshopper species, *K*. *tristis* has a single cul-de-sac spermatheca located at the end of a coiled spermathecal duct [36] (Muschett unpub. data.). Despite this common spermatheca morphology, the mechanisms of sperm precedence can vary widely among grasshopper species [37, 38]. For example, in *Chorthippus* grasshoppers, long intervals between mating events can result in a decline in the sperm numbers of the first male to mate, resulting in a large proportion of offspring sired by a subsequent mating male [37]. On the other hand, in *Locusta migratoria* a male’s spermatophore can act as a plug, blocking sperm transfer from succeeding males. When a female oviposits, the plug is ejected and any subsequent copulating male would be able to transfer sperm [83]. Future studies into the mechanism of spermatophore delivery and female treatment of the ejaculate in the spermatheca of *K*. *tristis* will provide important data toward understanding this process.

### Effect of female-male aggression on mating behaviour

*Kosciuscola tristis* females showed aggression towards males with occasional kicks and grapples. The frequency of female aggression also varied with the breeding season becoming marginally more frequent towards the end of the breeding season. However, female aggression occurred mainly before copulation and while this is likely to have some effect on male mating behaviour, we have no evidence that females can forcefully end copulation after it has begun. Female aggression occurs in other grasshopper species (e.g. *Gomphocerus rufus*, *Arphia pseudonietana*, *Shistocerca lineata*), [53] and [54] reported pre-copulatory female aggression towards males. Because many grasshopper species do not have a distinguishable courtship phase, communication between males and females typically occurs only once the male has mounted a female. The ‘struggling’ observed between a female and a mounted male in species like *Schistocerca* sp. and *G*. *rufus* occurs when a female is unreceptive or partially unreceptive [53, 54]. A similar process may occur in *K*. *tristis*, where a lack of obvious courtship combined with the high likelihood that late-season females have already mated, may consequently lead to more aggression towards males. Although recent evidence suggests rejecting multiple matings is counterproductive for females [84, 85, 86], in many grasshopper species subsequent copulations may offer no or very few additional fitness benefits as females typically obtain sufficient sperm to fertilize all their eggs from one mating [59, 87, 88]. It is possible that female *K*. *tristis* mate indiscriminately at the beginning of the breeding season in order to acquire sperm and become choosier as the breeding season progresses [89]. Alternatively, a female may struggle with a male until she receives the necessary stimuli from him, as occurs in some other grasshopper species (e.g. *Melanopus confuses*) where struggles can last for several minutes before copulation eventually ensues [53].

### Other variables affecting within-season mating and male-male aggressive behaviour

We do not know to what extent the changes in aggressive behaviour occurred within the same individuals or whether these behavioural changes reflect different cohorts of males. It is possible that not all males emerge as adults in synchrony and that there exists an early season cohort of non-fighters and a late season cohort of fighters. In other arthropod and grasshopper species (e.g. the grasshopper *Sphenarium purpurascens*), males that mature earlier usually achieve a larger body size and have greater fitness than those in cohorts that emerge later in the season [55, 90, 91]. However, early season *K*. *tristis* were no different in size to late season males, suggesting that there is only a single cohort of males. Further, we have never observed a sub-adult male after mid-February. Moreover, preliminary studies suggest that adult male *K*. *tristis* live at least 55 days [92] while other grasshopper species have been known to survive as adults up to 150+ days [93, 94]. Regardless, due to *K*. *tristis’* short life span and breeding season (a single alpine summer) it is likely there is strong selection on early synchronous hatching in this system, and there may not be enough suitable summer days to produce separate cohorts [88].

While we are aware that our results could be influenced by grasshopper age, the common method for determining age in Orthoptera (i.e. counting growth rings on the tibia) would not be possible in *K*. *tristis* because individuals of known age must be used for calibration [95]. Unfortunately, *K*. *tristis* are notoriously difficult to keep in captivity and have a high mortality rate [31]. There is strong evidence that *K*. *tristis* is protandrous and univoltine, with males emerging before females, within very a short period, and only one generation per year [30, 31, 42, 46]. The results of our study are consistent with this evidence, as we did not find size differences between males from the beginning and the end of the season. If all males are of a single cohort and thus, the same age, in Period 3 males will be older than in Period 1. It is possible there could be a reverse effect of age on aggression, with males being demonstrably more aggressive later in the season.

One potentially important factor affecting mating and aggressive behaviour not addressed in our study is the seasonal variation in the operational sex ratio. Because it describes the relative number of available mature males and females, the operational sex ratio is commonly used to predict the intensity of competition for mates [96]. However, the sex ratio in *K*. *tristis* is very difficult to determine in the field. Preliminary analyses yielded a male-biased sex ratio throughout the breeding season (S1 Table), but we are disinclined to use this data mainly due to observer bias. Bright turquoise males are more visible against the green/brown vegetation than the more cryptic greenish, brown females. In addition, males typically perch (bask) on the tops of alpine shrubs and grasses, while females typically perch in the lower parts of the vegetation (G. Muschett, K. Umbers pers. obs.). A reliable measure of the seasonal variation in the operational sex ratio remains a challenge for future studies on this species.

Finally, *K*. *tristis* is notable for the unique role that colour could play in aggressive interactions. While male colour/brightness is not an inter-sexual signal (females do not prefer brighter males), and neither does it correlate with other measures of quality such as body size, [31] suggests that males pay attention to each other’s brightness when deciding whether or not to enter antagonistic interactions. However, [31] found no evidence that brighter males win competitions, and the function of colour in this species is undetermined. Similarly, it is unlikely that the coloured bee tags and/or pen marks used in this study would affect male-male aggressive interactions.

### Conclusions

We demonstrate that the reproductive landscape of *K*. *tristis* varies significantly within the breeding season–males fight each other more often and more vigorously at the end of the season. Unexpectedly, female aggression seems to play an important role in this species’ mating system and merits further study. Our results suggest that cooler temperatures and increased late season female fecundity may act as cues for males to intensify intra-sexual aggression. As a semelparous species, there is probable selective pressure on *K*. *tristis* to time life cycle events with favourable biological and climatic conditions. The combination of a relatively short breeding season, limited mating opportunities toward the end of the season and lack of obvious courtship behaviour may have contributed to the evolution of this extraordinary mating system. While we are aware these experiments covered a single season, previous studies have reported that fighting does not occur until the peak of the breeding season [30, 31] and we are therefore confident that the variation in aggression is a seasonal occurrence. Our study provides further evidence for the seasonal nature of aggressive behaviour. While this variation in aggression could change depending on shifts in temperature or population density, this variability of aggressive behaviour is likely a common pattern across seasons. We recommend future research include multi-season comparisons.

## Supporting information

S1 Data Temperature data from the Bureau of Meteorology of Australia. Temperature data for the 2013–2014 growing/breeding season from the Thredbo Top Station (station number 071032).
(XLSX)

S2 Data Male-male aggression. Number of male-male aggressive behaviours per trial per period.
(XLSX)

S3 Data Female aggression. Number of female-male aggressive behaviours per trail per period.
(XLSX)

S4 Data Mating activity. Mating frequency (number of matings) per trial per period.
(XLSX)

S5 Data Latency to mate. Latency of first mating per trial per period.
(XLSX)

S6 Data Male and female size data. Correlation between mean male pronotum and femur length per trail and female size per trial.
(XLSX)

S7 Data Female weight. Female weight per trail per period.
(XLSX)

S8 Data Egg number. Number of mature ovarioles per female (per trial) per period.
(XLSX)

S9 Data Egg weight. Weight of mature ovarioles per female (per trial) per period.
(XLSX)

S1 Table Preliminary assessment of the operational sex ratio of *K*. *tristis* on Dead Horse Gap Trail, during Periods 1, 2, and 3. Mean number of adult *K*. *tristis* grasshoppers in a 1 x 1m quadrat during a 10min interval, at three different locations along Dead Horse Gap Trail during each of the sampling periods. M refers to males, F refers to females.
(XLSX)
